# *N*-(4-Meth­oxy­phen­yl)-6-methyl-2-phenyl-5-{[4-(tri­fluoro­meth­yl)anilino]meth­yl}pyrimidin-4-amine

**DOI:** 10.1107/S160053681303170X

**Published:** 2013-11-27

**Authors:** Jerzy Cieplik, Janusz Pluta, Iwona Bryndal, Tadeusz Lis

**Affiliations:** a Department of Organic Chemistry, Wrocław Medical University, 9 Grodzka St, 50-137 Wrocław, Poland; b Department of Applied Pharmacy, Wrocław Medical Uniwersity, 38 Szewska St, 50-137 Wrocław, Poland; c Department of Bioorganic Chemistry, Faculty of Engineering and Economics, Wrocław University of Economics, 118/120 Komandorska St, 53-345 Wrocław, Poland; d Faculty of Chemistry, University of Wrocław, 14 Joliot-Curie St, 50-383 Wrocław, Poland

## Abstract

The title compound, C_26_H_23_F_3_N_4_O, crystallizes with two symmetry-independent mol­ecules in the asymmetric unit, denoted *A* and *B*, which differ mainly in the rotation of the meth­oxy­phenyl ring. The –CF_3_ group of mol­ecule *B* is disordered by rotation, with the F atoms split over two sets of sites; the occupancy factor for the major component is 0.853 (4). The dihedral angles between the pyrimidine ring and the attached phenyl, meth­oxy­phenyl and tri­fluoro­methyl­phenyl rings are 8.1 (2), 37.5 (2) and 70.7 (2)°, respectively, in mol­ecule *A*, and 9.3 (2), 5.3 (2) and 79.7 (2)° in mol­ecule *B*. An intra­molecular N—H⋯N hydrogen bond occurs in each mol­ecule. In the crystal, two crystallographically independent mol­ecules associate into a dimer *via* a pair of N—H⋯N hydrogen bonds, with a resulting *R*_2_^2^(12) ring motif and π–π stacking inter­actions [centroid–centroid distance = 3.517 (4) Å] between the pyrimidine rings. For the *A* mol­ecules, there are inter­molecular C—H⋯O hydrogen bonds between an aryl C atom of meth­oxy­phenyl ring and a meth­oxy O atom of an adjacent mol­ecule. A similar inter­action is lacking in the *B* mol­ecules.

## Related literature

For the anti­bacterial activity of 6-methyl-2-phenyl-5-substituted pyrimidine derivatives, see: Cieplik *et al.* (1995 ▶, 2008 ▶). For related structures, see: Cieplik, Pluta *et al.* (2006 ▶, 2012 ▶); Cieplik, Stolarczyk *et al.* (2012 ▶).
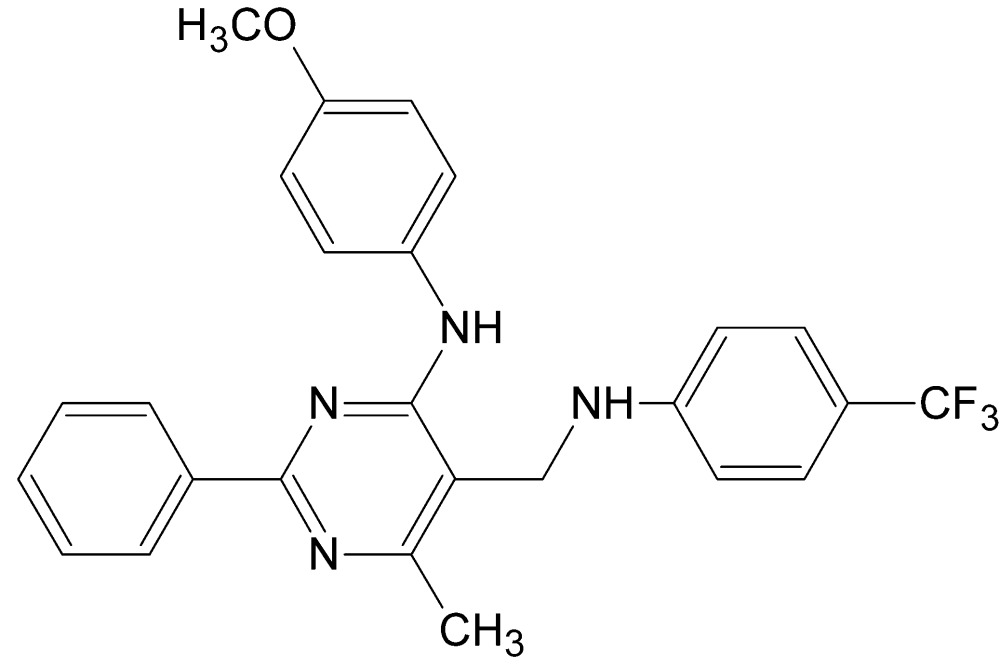


## Experimental

### 

#### Crystal data

C_26_H_23_F_3_N_4_O
*M* *_r_* = 464.48
Triclinic, 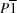

*a* = 8.724 (3) Å
*b* = 15.141 (6) Å
*c* = 17.844 (7) Å
α = 93.89 (3)°
β = 99.19 (3)°
γ = 103.26 (3)°
*V* = 2251.3 (15) Å^3^
*Z* = 4
Mo *K*α radiation
μ = 0.10 mm^−1^
*T* = 100 K
0.43 × 0.08 × 0.04 mm

#### Data collection

Oxford Diffraction Xcalibur PX diffractometer with Ruby CCD
19792 measured reflections
11372 independent reflections
8352 reflections with *I* > 2σ(*I*)
*R*_int_ = 0.022

#### Refinement

*R*[*F* ^2^ > 2σ(*F* ^2^)] = 0.054
*wR*(*F* ^2^) = 0.125
*S* = 1.03
11372 reflections
642 parameters
H atoms treated by a mixture of independent and constrained refinement
Δρ_max_ = 0.44 e Å^−3^
Δρ_min_ = −0.43 e Å^−3^

### 

Data collection: *CrysAlis CCD* (Oxford Diffraction, 2007 ▶); cell refinement: *CrysAlis RED* (Oxford Diffraction, 2007 ▶); data reduction: *CrysAlis RED*; program(s) used to solve structure: *SHELXS97* (Sheldrick, 2008 ▶); program(s) used to refine structure: *SHELXL97* (Sheldrick, 2008 ▶); molecular graphics: *DIAMOND* (Brandenburg, 2005) ▶; software used to prepare material for publication: *SHELXL97*.

## Supplementary Material

Crystal structure: contains datablock(s) I, New_Global_Publ_Block. DOI: 10.1107/S160053681303170X/nc2321sup1.cif

Structure factors: contains datablock(s) I. DOI: 10.1107/S160053681303170X/nc2321Isup2.hkl

Supplementary material file. DOI: 10.1107/S160053681303170X/nc2321Isup3.cml

Additional supplementary materials:  crystallographic information; 3D view; checkCIF report

## Figures and Tables

**Table 1:** Hydrogen-bond geometry (Å, °)

*D*—H⋯*A*	*D*—H	H⋯*A*	*D*⋯*A*	*D*—H⋯*A*
N5*A*—H5*A*⋯N1*B* ^i^	0.860 (19)	2.16 (2)	3.012 (2)	172.6 (17)
N5*B*—H5*B*⋯N3*A* ^i^	0.91 (2)	2.54 (2)	3.403 (2)	159.7 (17)
C43*A*—H43*A*⋯O4*A* ^ii^	0.95	2.45	3.355 (2)	159
N4*A*—H4*A*⋯N5*A*	0.86 (2)	2.48 (2)	3.099 (2)	130.2 (16)
N4*B*—H4*B*⋯N5*B*	0.87 (2)	2.31 (2)	3.021 (2)	139.0 (17)

Symmetry codes: (i) 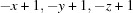; (ii) 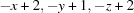.

**Fig. 1.**
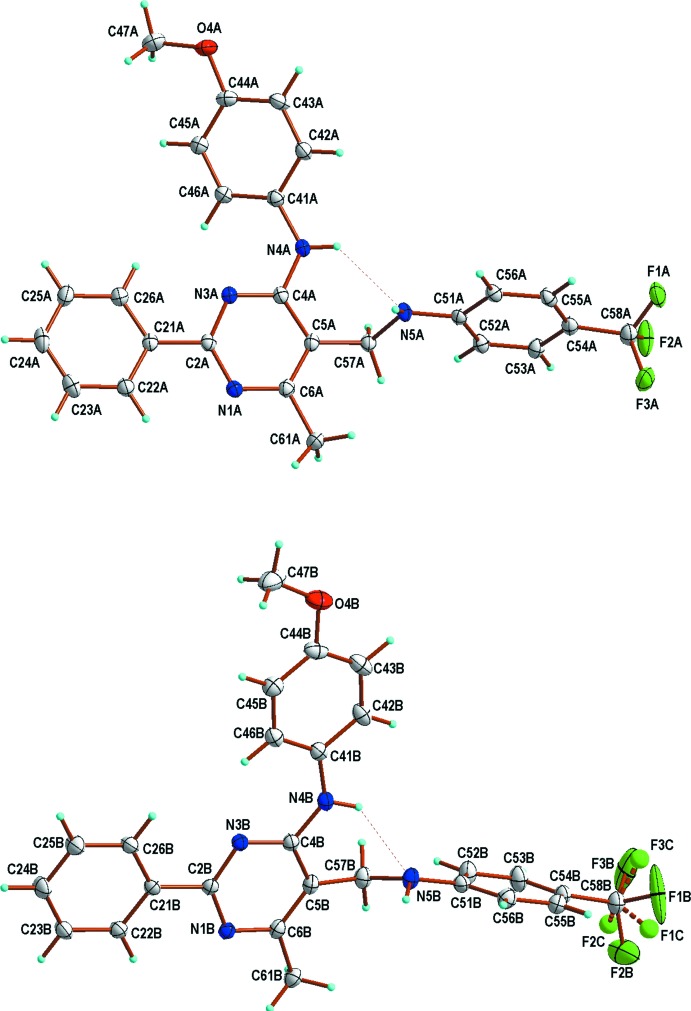
View of two symmetry independent molecules of the title compound with labelling scheme and displacement ellipsoids drawn at the 50% probability level. F atoms with the occupancy fator of 0.147 (4) and H atoms are shown as small spheres of arbitrary radii. The dotted lines indicate intramolecular N—H···O hydrogen bonds. Thick dashed lines represent fragment of a minor component of the disordered CF3 group.

**Fig. 2.**
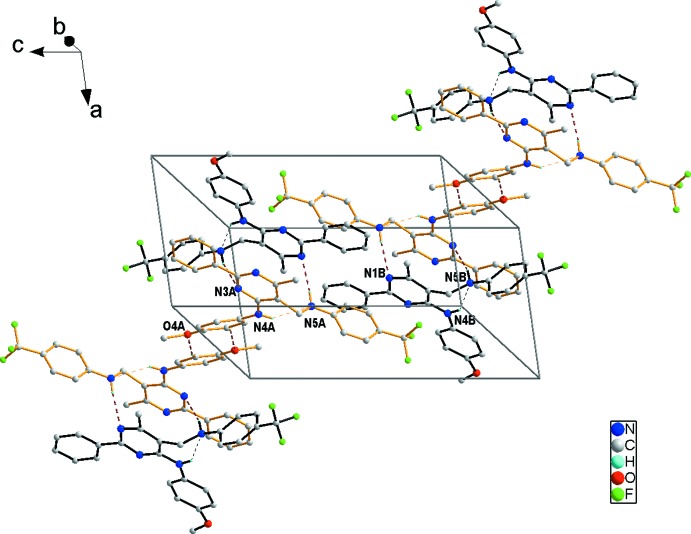
The arrangement of molecules A (light orange line) and B (black line), showing the intermolecular N—H···N and C—H···O hydrogen bonds (thick dashed lines). Thin dashed lines indicate intramolecular N—H···O interactions. The atoms of disordered CF3 group (with the smaller occupancy factor) and H atoms not involved in hydrogen bonding have been omitted for clarity.

## supplementary crystallographic information

### 1. Comment

The present paper is a continuation of our earlier works about the synthesis and biological activity of 6-methyl-2-phenyl-5-substitued pyrimidine derivatives (Cieplik *et al.*, 1995, 2008) as well as their structural data (Cieplik, Pluta *et al.*, 2006, 2012;Cieplik, Stolarczyk *et al.*, 2012).

The title compound, namely *N*-(4-methoxyphenyl)-6-methyl-2-phenyl-5-[(4-trifluoromethylanilino)methyl]pyrimidin-4-amine, crystallizes with two symmetry-independent molecules in the asymmetric unit, hereafter referred to as A and B (Fig. 1). The molecules differ in the orientation of the methoxyphenyl group with respect to the pyrimidine ring; the angle between the least-squares planes through the pyrimidine and aryl rings is 37.5 (2)° in molecule A and 5.3 (2)° in molecule B. For both molecules, the phenyl ring attached to the atom C2 is nearly co-planar with the pyrimidine ring [dihedral angle = 8.1 (2) and 9.3 (2)° in molecule A and B, respectively], whereas the trifluoromethylphenyl ring attached to the atom C5 is almost perpendicular to pyrimidine ring plane [dihedral angle = 70.7 (2) and 79.7 (2)° in molecule A and B, respectively].

In the crystal structure, the N5 amide atom of molecule A acts as hydrogen-bond donor to the pyrimidine atom N1 of molecule B at (-*x* + 1, -*y* + 1, -*z* + 1). Simultaneously, the amide atom N5 of molecule B acts as hydrogen-bond donor to the pyrimidine atom N3 of molecule A at (-*x* + 1, -*y* + 1, -*z* + 1). The result is the formation of a centrosymmetric hydrogen-bonded dimer with the *R*^2^_2_(12) ring motif. Furthemore, between pyrimidine rings of adjacent molecules within a dimer there is also an aromatic π-π stacking interaction (Fig. 2). The angle between the planes of these rings is 1.24 (9)°. The distance between the ring centroids of molecules at (*x*, *y*, *z*) and (-*x* + 1, -*y* + 1, -*z* + 1) is 3.517 (2) Å with an interplanar spacing of 3.488 (4) Å and a centroid offset of 0.45 Å. For molecules A, there are intermolecular C—H···O hydrogen bonds formed between the aryl atom C43A of the methoxyphenyl ring as a donor and the methoxy atom O4A at (-*x* + 2, -*y* + 1, -*z* + 2) as an acceptor (Fig. 2). A similar interaction is lacking in the B molecule. On the whole, a three-dimensional arrangment in the crystal structure consists of neighboring dimers, held together by C—H···O, C—F···π and C—H···π interactions as well as π–π interactions [the shortest centroid-centroid distance is 3.574 (4) Å].

### 2. Experimental

The title compound was obtained by adopting the procedure described previously by Cieplik *et al.* (1995). 4 g of 5-(chloromethyl)-*N*-(4-methoxyphenyl)-6-methyl-2-phenylpyrimidin-4-amine was dissolved in 30 ml of chloroform, and 3 g of 4-(trifluoromethyl)aniline. The reaction mixture was refluxed for 5 h with vigorous stirring, then was cooled and poured into 200 ml of water. The aqueous solution was extracted three times with chloroform (50 ml). The combined chloroform phases were dried over MgSO_4_, filtered and concentrated under vacuum. The oily residue was purified by column chromatography on silica gel (200–300 mesh) using CHCl_3_ as the eluent and by crystallization from methanol to give single crystals (yield: 4.3 g, 78.7%, m.p. 469–471 K).

### 3. Refinement

The C—H H atoms were positioned with idealized geometry (methyl H atoms allowed to rotate but not to tip) and were refined with *U*_iso_(H) = 1.2 *U*_eq_(C) (1.5 for methyl H atoms) using a riding model with C—H distances between 0.95–0.99 Å. The –CF_3_ group in molecule B is disordered with the F atoms split over two sets of sites and were refined with the occupancy factors of 0.853 (4) and 0.147 (4). The F atoms of higher occupancy were refined anisotropic, whereas those of lower occupancy were refined isotropic.

### Crystal data

**Table:**

C_26_H_23_F_3_N_4_O	*Z* = 4
*M**_r_* = 464.48	*F*(000) = 968
Triclinic, *P*1	*D*_x_ = 1.370 Mg m^−^^3^
Hall symbol: -P 1	Melting point: 470 K
*a* = 8.724 (3) Å	Mo *K*α radiation, λ = 0.71073 Å
*b* = 15.141 (6) Å	Cell parameters from 6466 reflections
*c* = 17.844 (7) Å	θ = 2.8–29.8°
α = 93.89 (3)°	µ = 0.10 mm^−^^1^
β = 99.19 (3)°	*T* = 100 K
γ = 103.26 (3)°	Plate, light yellow
*V* = 2251.3 (15) Å^3^	0.43 × 0.08 × 0.04 mm

### Data collection

**Table:**

Oxford Diffraction Xcalibur PX diffractometer with Ruby CCD	8352 reflections with *I* > 2σ(*I*)
Radiation source: fine-focus sealed tube	*R*_int_ = 0.022
Graphite monochromator	θ_max_ = 29.8°, θ_min_ = 2.8°
ω scans	*h* = −12→12
19792 measured reflections	*k* = −20→20
11372 independent reflections	*l* = −23→16

### Refinement

**Table:**

Refinement on *F*^2^	Primary atom site location: structure-invariant direct methods
Least-squares matrix: full	Secondary atom site location: difference Fourier map
*R*[*F*^2^ > 2σ(*F*^2^)] = 0.054	Hydrogen site location: inferred from neighbouring sites
*wR*(*F*^2^) = 0.125	H atoms treated by a mixture of independent and constrained refinement
*S* = 1.03	*w* = 1/[σ^2^(*F*_o_^2^) + (0.0405*P*)^2^ + 1.2149*P*] where *P* = (*F*_o_^2^ + 2*F*_c_^2^)/3
11372 reflections	(Δ/σ)_max_ = 0.001
642 parameters	Δρ_max_ = 0.44 e Å^−^^3^
0 restraints	Δρ_min_ = −0.43 e Å^−^^3^

### Special details

**Table:**

Geometry. All e.s.d.'s (except the e.s.d. in the dihedral angle between two l.s. planes) are estimated using the full covariance matrix. The cell e.s.d.'s are taken into account individually in the estimation of e.s.d.'s in distances, angles and torsion angles; correlations between e.s.d.'s in cell parameters are only used when they are defined by crystal symmetry. An approximate (isotropic) treatment of cell e.s.d.'s is used for estimating e.s.d.'s involving l.s. planes.
Refinement. Refinement of *F*^2^ against ALL reflections. The weighted *R*-factor *wR* and goodness of fit *S* are based on *F*^2^, conventional *R*-factors *R* are based on *F*, with *F* set to zero for negative *F*^2^. The threshold expression of *F*^2^ > σ(*F*^2^) is used only for calculating *R*-factors(gt) *etc*. and is not relevant to the choice of reflections for refinement. *R*-factors based on *F*^2^ are statistically about twice as large as those based on *F*, and *R*- factors based on ALL data will be even larger.

### Fractional atomic coordinates and isotropic or equivalent isotropic displacement parameters (Å^2^)

**Table:**

	*x*	*y*	*z*	*U*_iso_*/*U*_eq_	Occ. (<1)
N1A	0.76237 (16)	0.96728 (9)	0.70258 (8)	0.0196 (3)	
C2A	0.77090 (18)	0.93972 (10)	0.77236 (9)	0.0173 (3)	
C21A	0.72164 (19)	0.99489 (11)	0.83225 (9)	0.0185 (3)	
C22A	0.6485 (2)	1.06514 (11)	0.81371 (10)	0.0225 (3)	
H22A	0.6320	1.0792	0.7624	0.027*	
C23A	0.5994 (2)	1.11491 (12)	0.86999 (11)	0.0275 (4)	
H23A	0.5498	1.1628	0.8569	0.033*	
C24A	0.6225 (2)	1.09485 (12)	0.94470 (11)	0.0301 (4)	
H24A	0.5875	1.1283	0.9828	0.036*	
C25A	0.6969 (2)	1.02575 (12)	0.96409 (10)	0.0296 (4)	
H25A	0.7138	1.0122	1.0155	0.036*	
C26A	0.7467 (2)	0.97631 (11)	0.90814 (10)	0.0242 (4)	
H26A	0.7984	0.9294	0.9217	0.029*	
N3A	0.81915 (16)	0.86598 (9)	0.79446 (8)	0.0186 (3)	
C4A	0.87545 (19)	0.81943 (10)	0.74295 (9)	0.0183 (3)	
N4A	0.93179 (18)	0.74611 (10)	0.76491 (8)	0.0217 (3)	
H4A	0.939 (2)	0.7078 (13)	0.7290 (11)	0.026*	
C41A	0.95084 (19)	0.71744 (11)	0.83927 (9)	0.0198 (3)	
C42A	0.9273 (2)	0.62391 (11)	0.84616 (10)	0.0228 (3)	
H42A	0.8968	0.5814	0.8015	0.027*	
C43A	0.9480 (2)	0.59278 (12)	0.91726 (10)	0.0264 (4)	
H43A	0.9330	0.5292	0.9211	0.032*	
C44A	0.9908 (2)	0.65398 (12)	0.98311 (10)	0.0239 (4)	
C45A	1.0181 (2)	0.74671 (11)	0.97710 (10)	0.0226 (3)	
H45A	1.0500	0.7889	1.0219	0.027*	
C46A	0.9988 (2)	0.77814 (11)	0.90561 (9)	0.0214 (3)	
H46A	1.0186	0.8419	0.9020	0.026*	
O4A	1.00329 (17)	0.61577 (9)	1.05081 (7)	0.0336 (3)	
C47A	1.0558 (3)	0.67576 (14)	1.11979 (11)	0.0378 (5)	
H4A1	0.9799	0.7137	1.1243	0.057*	
H4A2	1.1618	0.7150	1.1192	0.057*	
H4A3	1.0620	0.6399	1.1634	0.057*	
C5A	0.87760 (19)	0.84423 (10)	0.66810 (9)	0.0180 (3)	
C57A	0.9519 (2)	0.79518 (11)	0.61278 (9)	0.0195 (3)	
H5A1	0.9597	0.8287	0.5673	0.023*	
H5A2	1.0617	0.7944	0.6372	0.023*	
N5A	0.85839 (17)	0.70154 (9)	0.58888 (8)	0.0178 (3)	
H5A	0.758 (2)	0.6910 (12)	0.5904 (10)	0.021*	
C51A	0.89123 (19)	0.65284 (11)	0.52727 (9)	0.0172 (3)	
C52A	1.02539 (19)	0.68484 (11)	0.49402 (9)	0.0197 (3)	
H52A	1.0987	0.7412	0.5145	0.024*	
C53A	1.0522 (2)	0.63490 (11)	0.43130 (9)	0.0214 (3)	
H53A	1.1426	0.6577	0.4086	0.026*	
C54A	0.9476 (2)	0.55191 (11)	0.40175 (9)	0.0209 (3)	
C55A	0.8172 (2)	0.51759 (11)	0.43617 (9)	0.0212 (3)	
H55A	0.7476	0.4597	0.4171	0.025*	
C56A	0.78866 (19)	0.56739 (11)	0.49788 (9)	0.0204 (3)	
H56A	0.6988	0.5437	0.5207	0.024*	
C58A	0.9720 (2)	0.50213 (12)	0.33149 (10)	0.0260 (4)	
F1A	0.91413 (14)	0.41104 (7)	0.32686 (6)	0.0357 (3)	
F2A	1.12666 (14)	0.51503 (9)	0.32574 (7)	0.0447 (3)	
F3A	0.89861 (17)	0.52706 (9)	0.26695 (6)	0.0454 (3)	
C6A	0.81483 (19)	0.91782 (11)	0.65045 (9)	0.0193 (3)	
C61A	0.8028 (2)	0.94979 (12)	0.57186 (10)	0.0277 (4)	
H6A1	0.9081	0.9851	0.5654	0.042*	
H6A2	0.7262	0.9883	0.5661	0.042*	
H6A3	0.7661	0.8968	0.5331	0.042*	
N1B	0.48661 (16)	0.31970 (9)	0.39572 (8)	0.0185 (3)	
C2B	0.54512 (18)	0.24961 (11)	0.41791 (9)	0.0171 (3)	
C21B	0.55299 (19)	0.23597 (11)	0.50022 (9)	0.0186 (3)	
C22B	0.5151 (2)	0.30014 (12)	0.54944 (9)	0.0227 (3)	
H22B	0.4793	0.3500	0.5295	0.027*	
C23B	0.5289 (2)	0.29203 (13)	0.62693 (10)	0.0280 (4)	
H23B	0.5032	0.3363	0.6598	0.034*	
C24B	0.5802 (2)	0.21937 (13)	0.65650 (10)	0.0315 (4)	
H24B	0.5903	0.2140	0.7097	0.038*	
C25B	0.6169 (2)	0.15432 (13)	0.60817 (10)	0.0297 (4)	
H25B	0.6514	0.1043	0.6283	0.036*	
C26B	0.6031 (2)	0.16238 (11)	0.53047 (10)	0.0229 (4)	
H26B	0.6278	0.1176	0.4977	0.028*	
N3B	0.59856 (16)	0.19190 (9)	0.37388 (7)	0.0184 (3)	
C4B	0.59796 (19)	0.20995 (11)	0.30120 (9)	0.0186 (3)	
N4B	0.64158 (17)	0.15329 (10)	0.25047 (8)	0.0220 (3)	
H4B	0.626 (2)	0.1677 (13)	0.2041 (12)	0.026*	
C41B	0.70212 (19)	0.07495 (11)	0.25837 (9)	0.0206 (3)	
C42B	0.7607 (2)	0.04472 (12)	0.19507 (10)	0.0261 (4)	
H42B	0.7578	0.0772	0.1514	0.031*	
C43B	0.8222 (2)	−0.03115 (12)	0.19513 (11)	0.0292 (4)	
H43B	0.8614	−0.0505	0.1517	0.035*	
C44B	0.8273 (2)	−0.07956 (12)	0.25850 (11)	0.0277 (4)	
C45B	0.7691 (2)	−0.05076 (12)	0.32163 (11)	0.0268 (4)	
H45B	0.7720	−0.0837	0.3651	0.032*	
C46B	0.7062 (2)	0.02651 (12)	0.32151 (10)	0.0236 (4)	
H46B	0.6664	0.0457	0.3648	0.028*	
O4B	0.88977 (17)	−0.15441 (9)	0.25234 (8)	0.0371 (3)	
C47B	0.9141 (3)	−0.20049 (15)	0.31864 (13)	0.0446 (6)	
H4B1	0.8111	−0.2238	0.3345	0.067*	
H4B2	0.9865	−0.1579	0.3600	0.067*	
H4B3	0.9613	−0.2515	0.3070	0.067*	
C5B	0.5562 (2)	0.28906 (11)	0.27414 (9)	0.0203 (3)	
C57B	0.5920 (2)	0.31925 (12)	0.19865 (9)	0.0247 (4)	
H5B1	0.5759	0.3815	0.1948	0.030*	
H5B2	0.7056	0.3218	0.1966	0.030*	
N5B	0.49060 (18)	0.25812 (10)	0.13371 (8)	0.0234 (3)	
H5B	0.393 (2)	0.2313 (13)	0.1434 (11)	0.028*	
C51B	0.4906 (2)	0.28915 (11)	0.06128 (9)	0.0218 (3)	
C52B	0.6123 (2)	0.36088 (13)	0.04864 (10)	0.0296 (4)	
H52B	0.6978	0.3891	0.0892	0.035*	
C53B	0.6088 (2)	0.39129 (13)	−0.02353 (10)	0.0310 (4)	
H53B	0.6910	0.4410	−0.0318	0.037*	
C54B	0.4866 (2)	0.34952 (12)	−0.08293 (9)	0.0235 (4)	
C55B	0.3669 (2)	0.27637 (12)	−0.07117 (10)	0.0259 (4)	
H55B	0.2835	0.2469	−0.1123	0.031*	
C56B	0.3694 (2)	0.24656 (12)	0.00045 (10)	0.0255 (4)	
H56B	0.2875	0.1965	0.0082	0.031*	
C58B	0.4802 (2)	0.38445 (14)	−0.15878 (10)	0.0305 (4)	
F1B	0.4052 (4)	0.31867 (17)	−0.21585 (9)	0.0929 (11)	0.853 (4)
F2B	0.3985 (4)	0.4458 (2)	−0.16732 (12)	0.1126 (15)	0.853 (4)
F3B	0.6199 (2)	0.4160 (2)	−0.17813 (11)	0.0692 (8)	0.853 (4)
F1C	0.3625 (10)	0.3680 (7)	−0.2021 (5)	0.032 (2)*	0.147 (4)
F2C	0.5351 (12)	0.4789 (6)	−0.1502 (5)	0.038 (3)*	0.147 (4)
F3C	0.5767 (19)	0.3600 (10)	−0.1951 (8)	0.071 (4)*	0.147 (4)
C6B	0.49560 (19)	0.34027 (11)	0.32344 (9)	0.0194 (3)	
C61B	0.4404 (2)	0.42421 (12)	0.30291 (10)	0.0259 (4)	
H6B1	0.5337	0.4758	0.3068	0.039*	
H6B2	0.3716	0.4382	0.3381	0.039*	
H6B3	0.3801	0.4132	0.2505	0.039*	

### Atomic displacement parameters (Å^2^)

**Table:**

	*U*^11^	*U*^22^	*U*^33^	*U*^12^	*U*^13^	*U*^23^
N1A	0.0220 (7)	0.0186 (7)	0.0189 (7)	0.0071 (6)	0.0037 (6)	0.0009 (5)
C2A	0.0169 (7)	0.0165 (7)	0.0177 (8)	0.0026 (6)	0.0036 (6)	0.0005 (6)
C21A	0.0185 (8)	0.0171 (7)	0.0194 (8)	0.0022 (6)	0.0058 (6)	−0.0005 (6)
C22A	0.0243 (8)	0.0207 (8)	0.0229 (8)	0.0065 (7)	0.0051 (7)	0.0003 (7)
C23A	0.0265 (9)	0.0232 (9)	0.0341 (10)	0.0096 (7)	0.0067 (8)	−0.0027 (8)
C24A	0.0318 (10)	0.0282 (9)	0.0310 (10)	0.0067 (8)	0.0135 (8)	−0.0080 (8)
C25A	0.0390 (11)	0.0292 (9)	0.0215 (9)	0.0074 (8)	0.0110 (8)	−0.0017 (7)
C26A	0.0316 (9)	0.0207 (8)	0.0219 (8)	0.0077 (7)	0.0073 (7)	0.0019 (7)
N3A	0.0223 (7)	0.0170 (6)	0.0172 (7)	0.0057 (5)	0.0046 (5)	0.0010 (5)
C4A	0.0197 (8)	0.0160 (7)	0.0189 (8)	0.0042 (6)	0.0034 (6)	−0.0001 (6)
N4A	0.0300 (8)	0.0194 (7)	0.0188 (7)	0.0112 (6)	0.0070 (6)	0.0009 (6)
C41A	0.0196 (8)	0.0203 (8)	0.0216 (8)	0.0073 (6)	0.0050 (6)	0.0040 (6)
C42A	0.0251 (9)	0.0186 (8)	0.0248 (9)	0.0075 (7)	0.0027 (7)	0.0003 (7)
C43A	0.0307 (9)	0.0168 (8)	0.0314 (10)	0.0070 (7)	0.0013 (8)	0.0059 (7)
C44A	0.0246 (9)	0.0243 (8)	0.0250 (9)	0.0080 (7)	0.0047 (7)	0.0104 (7)
C45A	0.0265 (9)	0.0220 (8)	0.0197 (8)	0.0070 (7)	0.0039 (7)	0.0017 (7)
C46A	0.0257 (9)	0.0176 (8)	0.0222 (8)	0.0066 (7)	0.0053 (7)	0.0033 (6)
O4A	0.0495 (9)	0.0272 (7)	0.0250 (7)	0.0104 (6)	0.0042 (6)	0.0112 (5)
C47A	0.0551 (13)	0.0376 (11)	0.0242 (10)	0.0178 (10)	0.0052 (9)	0.0098 (8)
C5A	0.0190 (8)	0.0174 (7)	0.0165 (7)	0.0026 (6)	0.0041 (6)	−0.0017 (6)
C57A	0.0217 (8)	0.0182 (8)	0.0188 (8)	0.0042 (6)	0.0062 (6)	−0.0002 (6)
N5A	0.0159 (6)	0.0177 (6)	0.0198 (7)	0.0037 (5)	0.0051 (6)	−0.0013 (5)
C51A	0.0184 (7)	0.0186 (7)	0.0162 (7)	0.0088 (6)	0.0013 (6)	0.0012 (6)
C52A	0.0174 (8)	0.0197 (8)	0.0214 (8)	0.0053 (6)	0.0022 (6)	−0.0007 (6)
C53A	0.0205 (8)	0.0248 (8)	0.0200 (8)	0.0074 (7)	0.0046 (7)	0.0000 (7)
C54A	0.0222 (8)	0.0233 (8)	0.0179 (8)	0.0114 (7)	−0.0012 (7)	−0.0012 (6)
C55A	0.0221 (8)	0.0174 (8)	0.0217 (8)	0.0055 (6)	−0.0022 (7)	−0.0021 (6)
C56A	0.0189 (8)	0.0202 (8)	0.0223 (8)	0.0057 (6)	0.0035 (7)	0.0016 (7)
C58A	0.0263 (9)	0.0298 (9)	0.0229 (9)	0.0123 (7)	0.0013 (7)	−0.0027 (7)
F1A	0.0466 (7)	0.0272 (6)	0.0321 (6)	0.0128 (5)	0.0037 (5)	−0.0099 (5)
F2A	0.0304 (6)	0.0562 (8)	0.0449 (7)	0.0093 (6)	0.0127 (5)	−0.0229 (6)
F3A	0.0727 (9)	0.0526 (8)	0.0187 (5)	0.0369 (7)	0.0015 (6)	−0.0002 (5)
C6A	0.0204 (8)	0.0186 (8)	0.0173 (8)	0.0028 (6)	0.0031 (6)	−0.0005 (6)
C61A	0.0406 (11)	0.0285 (9)	0.0176 (8)	0.0148 (8)	0.0063 (8)	0.0037 (7)
N1B	0.0168 (6)	0.0215 (7)	0.0181 (7)	0.0056 (5)	0.0039 (5)	0.0050 (5)
C2B	0.0132 (7)	0.0204 (8)	0.0172 (8)	0.0023 (6)	0.0032 (6)	0.0035 (6)
C21B	0.0169 (7)	0.0219 (8)	0.0183 (8)	0.0050 (6)	0.0056 (6)	0.0042 (6)
C22B	0.0273 (9)	0.0238 (8)	0.0216 (8)	0.0121 (7)	0.0079 (7)	0.0066 (7)
C23B	0.0396 (11)	0.0301 (9)	0.0211 (9)	0.0166 (8)	0.0125 (8)	0.0045 (7)
C24B	0.0480 (12)	0.0355 (10)	0.0198 (9)	0.0195 (9)	0.0151 (8)	0.0116 (8)
C25B	0.0437 (11)	0.0287 (9)	0.0255 (9)	0.0189 (8)	0.0137 (8)	0.0123 (8)
C26B	0.0291 (9)	0.0227 (8)	0.0221 (8)	0.0118 (7)	0.0105 (7)	0.0055 (7)
N3B	0.0180 (7)	0.0206 (7)	0.0169 (7)	0.0048 (5)	0.0036 (5)	0.0029 (5)
C4B	0.0162 (7)	0.0216 (8)	0.0169 (8)	0.0028 (6)	0.0029 (6)	0.0013 (6)
N4B	0.0259 (8)	0.0247 (7)	0.0163 (7)	0.0070 (6)	0.0051 (6)	0.0033 (6)
C41B	0.0178 (8)	0.0213 (8)	0.0210 (8)	0.0025 (6)	0.0035 (6)	−0.0014 (7)
C42B	0.0265 (9)	0.0265 (9)	0.0242 (9)	0.0017 (7)	0.0094 (7)	−0.0003 (7)
C43B	0.0271 (9)	0.0271 (9)	0.0326 (10)	0.0031 (7)	0.0124 (8)	−0.0060 (8)
C44B	0.0222 (9)	0.0213 (8)	0.0376 (10)	0.0049 (7)	0.0035 (8)	−0.0043 (8)
C45B	0.0269 (9)	0.0248 (9)	0.0277 (9)	0.0057 (7)	0.0029 (8)	0.0031 (7)
C46B	0.0236 (9)	0.0247 (8)	0.0216 (8)	0.0042 (7)	0.0049 (7)	0.0000 (7)
O4B	0.0387 (8)	0.0280 (7)	0.0479 (9)	0.0147 (6)	0.0100 (7)	−0.0011 (6)
C47B	0.0470 (13)	0.0369 (11)	0.0510 (14)	0.0230 (10)	−0.0045 (11)	−0.0005 (10)
C5B	0.0209 (8)	0.0231 (8)	0.0155 (8)	0.0032 (7)	0.0020 (6)	0.0034 (6)
C57B	0.0261 (9)	0.0265 (9)	0.0205 (8)	0.0037 (7)	0.0046 (7)	0.0049 (7)
N5B	0.0235 (7)	0.0269 (8)	0.0184 (7)	0.0026 (6)	0.0035 (6)	0.0049 (6)
C51B	0.0253 (9)	0.0248 (8)	0.0193 (8)	0.0109 (7)	0.0075 (7)	0.0060 (7)
C52B	0.0289 (10)	0.0368 (10)	0.0192 (9)	0.0025 (8)	0.0015 (7)	0.0024 (8)
C53B	0.0347 (10)	0.0356 (10)	0.0217 (9)	0.0030 (8)	0.0090 (8)	0.0069 (8)
C54B	0.0305 (9)	0.0276 (9)	0.0171 (8)	0.0126 (7)	0.0082 (7)	0.0066 (7)
C55B	0.0277 (9)	0.0305 (9)	0.0211 (8)	0.0114 (8)	0.0031 (7)	0.0029 (7)
C56B	0.0258 (9)	0.0258 (9)	0.0249 (9)	0.0053 (7)	0.0046 (7)	0.0055 (7)
C58B	0.0378 (11)	0.0373 (11)	0.0196 (9)	0.0136 (9)	0.0066 (8)	0.0066 (8)
F1B	0.153 (2)	0.0736 (16)	0.0148 (8)	−0.0459 (16)	0.0155 (10)	−0.0035 (8)
F2B	0.191 (3)	0.169 (3)	0.0605 (13)	0.157 (3)	0.0730 (17)	0.0816 (16)
F3B	0.0387 (9)	0.116 (2)	0.0414 (10)	−0.0134 (11)	0.0035 (8)	0.0516 (12)
C6B	0.0171 (8)	0.0214 (8)	0.0182 (8)	0.0023 (6)	0.0012 (6)	0.0045 (6)
C61B	0.0295 (9)	0.0292 (9)	0.0228 (9)	0.0128 (8)	0.0051 (7)	0.0101 (7)

### Geometric parameters (Å, º)

**Table:**

N1A—C2A	1.336 (2)	C2B—N3B	1.345 (2)
N1A—C6A	1.354 (2)	C2B—C21B	1.490 (2)
C2A—N3A	1.342 (2)	C21B—C22B	1.396 (2)
C2A—C21A	1.488 (2)	C21B—C26B	1.398 (2)
C21A—C22A	1.393 (2)	C22B—C23B	1.385 (2)
C21A—C26A	1.395 (2)	C22B—H22B	0.9500
C22A—C23A	1.394 (2)	C23B—C24B	1.385 (2)
C22A—H22A	0.9500	C23B—H23B	0.9500
C23A—C24A	1.381 (3)	C24B—C25B	1.391 (3)
C23A—H23A	0.9500	C24B—H24B	0.9500
C24A—C25A	1.387 (3)	C25B—C26B	1.388 (2)
C24A—H24A	0.9500	C25B—H25B	0.9500
C25A—C26A	1.390 (2)	C26B—H26B	0.9500
C25A—H25A	0.9500	N3B—C4B	1.343 (2)
C26A—H26A	0.9500	C4B—N4B	1.362 (2)
N3A—C4A	1.341 (2)	C4B—C5B	1.422 (2)
C4A—N4A	1.370 (2)	N4B—C41B	1.411 (2)
C4A—C5A	1.414 (2)	N4B—H4B	0.87 (2)
N4A—C41A	1.420 (2)	C41B—C46B	1.385 (2)
N4A—H4A	0.86 (2)	C41B—C42B	1.403 (2)
C41A—C46A	1.392 (2)	C42B—C43B	1.375 (3)
C41A—C42A	1.400 (2)	C42B—H42B	0.9500
C42A—C43A	1.383 (2)	C43B—C44B	1.389 (3)
C42A—H42A	0.9500	C43B—H43B	0.9500
C43A—C44A	1.390 (3)	C44B—O4B	1.371 (2)
C43A—H43A	0.9500	C44B—C45B	1.391 (3)
C44A—O4A	1.374 (2)	C45B—C46B	1.402 (2)
C44A—C45A	1.384 (2)	C45B—H45B	0.9500
C45A—C46A	1.390 (2)	C46B—H46B	0.9500
C45A—H45A	0.9500	O4B—C47B	1.425 (3)
C46A—H46A	0.9500	C47B—H4B1	0.9800
O4A—C47A	1.424 (2)	C47B—H4B2	0.9800
C47A—H4A1	0.9800	C47B—H4B3	0.9800
C47A—H4A2	0.9800	C5B—C6B	1.378 (2)
C47A—H4A3	0.9800	C5B—C57B	1.508 (2)
C5A—C6A	1.383 (2)	C57B—N5B	1.461 (2)
C5A—C57A	1.507 (2)	C57B—H5B1	0.9900
C57A—N5A	1.461 (2)	C57B—H5B2	0.9900
C57A—H5A1	0.9900	N5B—C51B	1.405 (2)
C57A—H5A2	0.9900	N5B—H5B	0.91 (2)
N5A—C51A	1.386 (2)	C51B—C56B	1.392 (3)
N5A—H5A	0.860 (19)	C51B—C52B	1.392 (3)
C51A—C52A	1.399 (2)	C52B—C53B	1.395 (3)
C51A—C56A	1.406 (2)	C52B—H52B	0.9500
C52A—C53A	1.389 (2)	C53B—C54B	1.378 (3)
C52A—H52A	0.9500	C53B—H53B	0.9500
C53A—C54A	1.386 (2)	C54B—C55B	1.390 (3)
C53A—H53A	0.9500	C54B—C58B	1.484 (2)
C54A—C55A	1.392 (2)	C55B—C56B	1.382 (2)
C54A—C58A	1.489 (2)	C55B—H55B	0.9500
C55A—C56A	1.380 (2)	C56B—H56B	0.9500
C55A—H55A	0.9500	C58B—F1C	1.147 (9)
C56A—H56A	0.9500	C58B—F3C	1.245 (13)
C58A—F2A	1.340 (2)	C58B—F2B	1.297 (3)
C58A—F3A	1.347 (2)	C58B—F3B	1.312 (3)
C58A—F1A	1.348 (2)	C58B—F1B	1.345 (3)
C6A—C61A	1.510 (2)	C58B—F2C	1.390 (8)
C61A—H6A1	0.9800	C6B—C61B	1.506 (2)
C61A—H6A2	0.9800	C61B—H6B1	0.9800
C61A—H6A3	0.9800	C61B—H6B2	0.9800
N1B—C2B	1.336 (2)	C61B—H6B3	0.9800
N1B—C6B	1.357 (2)		
			
C2A—N1A—C6A	116.17 (14)	N3B—C2B—C21B	117.67 (14)
N1A—C2A—N3A	126.42 (14)	C22B—C21B—C26B	118.70 (15)
N1A—C2A—C21A	117.79 (14)	C22B—C21B—C2B	119.33 (14)
N3A—C2A—C21A	115.80 (14)	C26B—C21B—C2B	121.93 (14)
C22A—C21A—C26A	118.80 (15)	C23B—C22B—C21B	120.84 (15)
C22A—C21A—C2A	120.90 (15)	C23B—C22B—H22B	119.6
C26A—C21A—C2A	120.30 (15)	C21B—C22B—H22B	119.6
C21A—C22A—C23A	120.38 (16)	C22B—C23B—C24B	120.04 (16)
C21A—C22A—H22A	119.8	C22B—C23B—H23B	120.0
C23A—C22A—H22A	119.8	C24B—C23B—H23B	120.0
C24A—C23A—C22A	120.27 (17)	C23B—C24B—C25B	119.87 (16)
C24A—C23A—H23A	119.9	C23B—C24B—H24B	120.1
C22A—C23A—H23A	119.9	C25B—C24B—H24B	120.1
C23A—C24A—C25A	119.93 (16)	C26B—C25B—C24B	120.14 (16)
C23A—C24A—H24A	120.0	C26B—C25B—H25B	119.9
C25A—C24A—H24A	120.0	C24B—C25B—H25B	119.9
C24A—C25A—C26A	119.92 (17)	C25B—C26B—C21B	120.41 (15)
C24A—C25A—H25A	120.0	C25B—C26B—H26B	119.8
C26A—C25A—H25A	120.0	C21B—C26B—H26B	119.8
C25A—C26A—C21A	120.70 (16)	C4B—N3B—C2B	115.63 (14)
C25A—C26A—H26A	119.7	N3B—C4B—N4B	120.65 (15)
C21A—C26A—H26A	119.7	N3B—C4B—C5B	122.21 (14)
C4A—N3A—C2A	116.58 (14)	N4B—C4B—C5B	117.12 (15)
N3A—C4A—N4A	117.90 (15)	C4B—N4B—C41B	132.62 (15)
N3A—C4A—C5A	121.85 (14)	C4B—N4B—H4B	112.9 (13)
N4A—C4A—C5A	120.26 (14)	C41B—N4B—H4B	114.5 (13)
C4A—N4A—C41A	127.03 (14)	C46B—C41B—C42B	118.77 (16)
C4A—N4A—H4A	116.4 (13)	C46B—C41B—N4B	125.95 (15)
C41A—N4A—H4A	115.5 (13)	C42B—C41B—N4B	115.27 (15)
C46A—C41A—C42A	118.36 (15)	C43B—C42B—C41B	121.18 (17)
C46A—C41A—N4A	123.10 (15)	C43B—C42B—H42B	119.4
C42A—C41A—N4A	118.48 (15)	C41B—C42B—H42B	119.4
C43A—C42A—C41A	120.64 (16)	C42B—C43B—C44B	120.17 (17)
C43A—C42A—H42A	119.7	C42B—C43B—H43B	119.9
C41A—C42A—H42A	119.7	C44B—C43B—H43B	119.9
C42A—C43A—C44A	120.37 (16)	O4B—C44B—C43B	115.42 (16)
C42A—C43A—H43A	119.8	O4B—C44B—C45B	125.17 (18)
C44A—C43A—H43A	119.8	C43B—C44B—C45B	119.41 (16)
O4A—C44A—C45A	124.72 (16)	C44B—C45B—C46B	120.39 (17)
O4A—C44A—C43A	115.69 (15)	C44B—C45B—H45B	119.8
C45A—C44A—C43A	119.58 (16)	C46B—C45B—H45B	119.8
C44A—C45A—C46A	120.07 (16)	C41B—C46B—C45B	120.07 (16)
C44A—C45A—H45A	120.0	C41B—C46B—H46B	120.0
C46A—C45A—H45A	120.0	C45B—C46B—H46B	120.0
C45A—C46A—C41A	120.93 (15)	C44B—O4B—C47B	117.60 (16)
C45A—C46A—H46A	119.5	O4B—C47B—H4B1	109.5
C41A—C46A—H46A	119.5	O4B—C47B—H4B2	109.5
C44A—O4A—C47A	117.84 (14)	H4B1—C47B—H4B2	109.5
O4A—C47A—H4A1	109.5	O4B—C47B—H4B3	109.5
O4A—C47A—H4A2	109.5	H4B1—C47B—H4B3	109.5
H4A1—C47A—H4A2	109.5	H4B2—C47B—H4B3	109.5
O4A—C47A—H4A3	109.5	C6B—C5B—C4B	116.38 (15)
H4A1—C47A—H4A3	109.5	C6B—C5B—C57B	122.28 (15)
H4A2—C47A—H4A3	109.5	C4B—C5B—C57B	121.08 (15)
C6A—C5A—C4A	116.23 (14)	N5B—C57B—C5B	112.42 (14)
C6A—C5A—C57A	122.67 (15)	N5B—C57B—H5B1	109.1
C4A—C5A—C57A	121.03 (14)	C5B—C57B—H5B1	109.1
N5A—C57A—C5A	111.76 (14)	N5B—C57B—H5B2	109.1
N5A—C57A—H5A1	109.3	C5B—C57B—H5B2	109.1
C5A—C57A—H5A1	109.3	H5B1—C57B—H5B2	107.9
N5A—C57A—H5A2	109.3	C51B—N5B—C57B	117.00 (14)
C5A—C57A—H5A2	109.3	C51B—N5B—H5B	115.1 (13)
H5A1—C57A—H5A2	107.9	C57B—N5B—H5B	112.8 (12)
C51A—N5A—C57A	119.11 (14)	C56B—C51B—C52B	119.07 (16)
C51A—N5A—H5A	113.0 (12)	C56B—C51B—N5B	119.87 (16)
C57A—N5A—H5A	116.4 (12)	C52B—C51B—N5B	121.05 (16)
N5A—C51A—C52A	122.27 (15)	C51B—C52B—C53B	120.07 (17)
N5A—C51A—C56A	119.24 (15)	C51B—C52B—H52B	120.0
C52A—C51A—C56A	118.47 (14)	C53B—C52B—H52B	120.0
C53A—C52A—C51A	120.50 (15)	C54B—C53B—C52B	120.23 (18)
C53A—C52A—H52A	119.7	C54B—C53B—H53B	119.9
C51A—C52A—H52A	119.7	C52B—C53B—H53B	119.9
C54A—C53A—C52A	120.26 (16)	C53B—C54B—C55B	119.99 (16)
C54A—C53A—H53A	119.9	C53B—C54B—C58B	119.97 (17)
C52A—C53A—H53A	119.9	C55B—C54B—C58B	120.02 (17)
C53A—C54A—C55A	119.80 (15)	C56B—C55B—C54B	119.89 (17)
C53A—C54A—C58A	119.59 (16)	C56B—C55B—H55B	120.1
C55A—C54A—C58A	120.56 (16)	C54B—C55B—H55B	120.1
C56A—C55A—C54A	120.23 (16)	C55B—C56B—C51B	120.72 (17)
C56A—C55A—H55A	119.9	C55B—C56B—H56B	119.6
C54A—C55A—H55A	119.9	C51B—C56B—H56B	119.6
C55A—C56A—C51A	120.66 (16)	F1C—C58B—F3C	103.9 (8)
C55A—C56A—H56A	119.7	F2B—C58B—F3B	108.4 (2)
C51A—C56A—H56A	119.7	F2B—C58B—F1B	103.4 (2)
F2A—C58A—F3A	106.24 (15)	F3B—C58B—F1B	103.09 (19)
F2A—C58A—F1A	105.81 (14)	F1C—C58B—F2C	106.0 (6)
F3A—C58A—F1A	104.94 (14)	F3C—C58B—F2C	102.1 (8)
F2A—C58A—C54A	112.95 (15)	F1C—C58B—C54B	120.7 (4)
F3A—C58A—C54A	112.84 (14)	F3C—C58B—C54B	112.0 (6)
F1A—C58A—C54A	113.37 (16)	F2B—C58B—C54B	113.64 (16)
N1A—C6A—C5A	122.52 (15)	F3B—C58B—C54B	115.09 (17)
N1A—C6A—C61A	115.20 (14)	F1B—C58B—C54B	112.06 (18)
C5A—C6A—C61A	122.27 (14)	F2C—C58B—C54B	110.3 (4)
C6A—C61A—H6A1	109.5	N1B—C6B—C5B	121.65 (15)
C6A—C61A—H6A2	109.5	N1B—C6B—C61B	115.08 (14)
H6A1—C61A—H6A2	109.5	C5B—C6B—C61B	123.23 (15)
C6A—C61A—H6A3	109.5	C6B—C61B—H6B1	109.5
H6A1—C61A—H6A3	109.5	C6B—C61B—H6B2	109.5
H6A2—C61A—H6A3	109.5	H6B1—C61B—H6B2	109.5
C2B—N1B—C6B	116.87 (14)	C6B—C61B—H6B3	109.5
N1B—C2B—N3B	126.61 (15)	H6B1—C61B—H6B3	109.5
N1B—C2B—C21B	115.72 (14)	H6B2—C61B—H6B3	109.5
			
C6A—N1A—C2A—N3A	−3.3 (2)	N3B—C2B—C21B—C22B	173.11 (15)
C6A—N1A—C2A—C21A	177.16 (14)	N1B—C2B—C21B—C26B	176.34 (15)
N1A—C2A—C21A—C22A	8.7 (2)	N3B—C2B—C21B—C26B	−4.5 (2)
N3A—C2A—C21A—C22A	−170.91 (15)	C26B—C21B—C22B—C23B	0.9 (3)
N1A—C2A—C21A—C26A	−171.94 (15)	C2B—C21B—C22B—C23B	−176.72 (16)
N3A—C2A—C21A—C26A	8.4 (2)	C21B—C22B—C23B—C24B	−0.3 (3)
C26A—C21A—C22A—C23A	−0.9 (3)	C22B—C23B—C24B—C25B	−0.4 (3)
C2A—C21A—C22A—C23A	178.45 (16)	C23B—C24B—C25B—C26B	0.4 (3)
C21A—C22A—C23A—C24A	−0.2 (3)	C24B—C25B—C26B—C21B	0.3 (3)
C22A—C23A—C24A—C25A	0.9 (3)	C22B—C21B—C26B—C25B	−0.9 (3)
C23A—C24A—C25A—C26A	−0.6 (3)	C2B—C21B—C26B—C25B	176.66 (16)
C24A—C25A—C26A—C21A	−0.5 (3)	N1B—C2B—N3B—C4B	2.8 (2)
C22A—C21A—C26A—C25A	1.2 (3)	C21B—C2B—N3B—C4B	−176.25 (14)
C2A—C21A—C26A—C25A	−178.12 (16)	C2B—N3B—C4B—N4B	−176.55 (14)
N1A—C2A—N3A—C4A	5.3 (2)	C2B—N3B—C4B—C5B	5.0 (2)
C21A—C2A—N3A—C4A	−175.08 (14)	N3B—C4B—N4B—C41B	−4.7 (3)
C2A—N3A—C4A—N4A	177.33 (14)	C5B—C4B—N4B—C41B	173.80 (16)
C2A—N3A—C4A—C5A	−2.7 (2)	C4B—N4B—C41B—C46B	12.1 (3)
N3A—C4A—N4A—C41A	−5.9 (3)	C4B—N4B—C41B—C42B	−168.68 (17)
C5A—C4A—N4A—C41A	174.03 (16)	C46B—C41B—C42B—C43B	−0.4 (3)
C4A—N4A—C41A—C46A	−36.2 (3)	N4B—C41B—C42B—C43B	−179.65 (16)
C4A—N4A—C41A—C42A	146.82 (17)	C41B—C42B—C43B—C44B	0.1 (3)
C46A—C41A—C42A—C43A	1.3 (2)	C42B—C43B—C44B—O4B	179.52 (16)
N4A—C41A—C42A—C43A	178.51 (16)	C42B—C43B—C44B—C45B	0.2 (3)
C41A—C42A—C43A—C44A	0.8 (3)	O4B—C44B—C45B—C46B	−179.38 (16)
C42A—C43A—C44A—O4A	178.00 (16)	C43B—C44B—C45B—C46B	−0.1 (3)
C42A—C43A—C44A—C45A	−2.3 (3)	C42B—C41B—C46B—C45B	0.5 (2)
O4A—C44A—C45A—C46A	−178.66 (16)	N4B—C41B—C46B—C45B	179.63 (16)
C43A—C44A—C45A—C46A	1.6 (3)	C44B—C45B—C46B—C41B	−0.2 (3)
C44A—C45A—C46A—C41A	0.5 (3)	C43B—C44B—O4B—C47B	173.20 (17)
C42A—C41A—C46A—C45A	−2.0 (2)	C45B—C44B—O4B—C47B	−7.5 (3)
N4A—C41A—C46A—C45A	−179.01 (16)	N3B—C4B—C5B—C6B	−8.4 (2)
C45A—C44A—O4A—C47A	−3.3 (3)	N4B—C4B—C5B—C6B	173.10 (15)
C43A—C44A—O4A—C47A	176.40 (17)	N3B—C4B—C5B—C57B	165.89 (15)
N3A—C4A—C5A—C6A	−1.5 (2)	N4B—C4B—C5B—C57B	−12.6 (2)
N4A—C4A—C5A—C6A	178.51 (15)	C6B—C5B—C57B—N5B	−115.30 (18)
N3A—C4A—C5A—C57A	175.67 (15)	C4B—C5B—C57B—N5B	70.8 (2)
N4A—C4A—C5A—C57A	−4.3 (2)	C5B—C57B—N5B—C51B	167.43 (14)
C6A—C5A—C57A—N5A	−113.92 (17)	C57B—N5B—C51B—C56B	−163.61 (15)
C4A—C5A—C57A—N5A	69.1 (2)	C57B—N5B—C51B—C52B	17.9 (2)
C5A—C57A—N5A—C51A	167.33 (13)	C56B—C51B—C52B—C53B	2.2 (3)
C57A—N5A—C51A—C52A	9.6 (2)	N5B—C51B—C52B—C53B	−179.23 (17)
C57A—N5A—C51A—C56A	−171.81 (14)	C51B—C52B—C53B—C54B	−1.1 (3)
N5A—C51A—C52A—C53A	−178.62 (15)	C52B—C53B—C54B—C55B	−0.5 (3)
C56A—C51A—C52A—C53A	2.8 (2)	C52B—C53B—C54B—C58B	177.63 (17)
C51A—C52A—C53A—C54A	−1.1 (2)	C53B—C54B—C55B—C56B	1.0 (3)
C52A—C53A—C54A—C55A	−1.5 (2)	C58B—C54B—C55B—C56B	−177.13 (16)
C52A—C53A—C54A—C58A	176.02 (15)	C54B—C55B—C56B—C51B	0.2 (3)
C53A—C54A—C55A—C56A	2.3 (2)	C52B—C51B—C56B—C55B	−1.8 (3)
C58A—C54A—C55A—C56A	−175.15 (15)	N5B—C51B—C56B—C55B	179.70 (15)
C54A—C55A—C56A—C51A	−0.6 (2)	C53B—C54B—C58B—F1C	−159.3 (6)
N5A—C51A—C56A—C55A	179.41 (14)	C55B—C54B—C58B—F1C	18.8 (7)
C52A—C51A—C56A—C55A	−1.9 (2)	C53B—C54B—C58B—F3C	77.9 (8)
C53A—C54A—C58A—F2A	32.5 (2)	C55B—C54B—C58B—F3C	−104.0 (8)
C55A—C54A—C58A—F2A	−150.03 (16)	C53B—C54B—C58B—F2B	−90.5 (3)
C53A—C54A—C58A—F3A	−88.0 (2)	C55B—C54B—C58B—F2B	87.6 (3)
C55A—C54A—C58A—F3A	89.5 (2)	C53B—C54B—C58B—F3B	35.3 (3)
C53A—C54A—C58A—F1A	152.88 (15)	C55B—C54B—C58B—F3B	−146.6 (2)
C55A—C54A—C58A—F1A	−29.7 (2)	C53B—C54B—C58B—F1B	152.7 (2)
C2A—N1A—C6A—C5A	−1.6 (2)	C55B—C54B—C58B—F1B	−29.2 (3)
C2A—N1A—C6A—C61A	179.52 (15)	C53B—C54B—C58B—F2C	−35.1 (5)
C4A—C5A—C6A—N1A	3.7 (2)	C55B—C54B—C58B—F2C	143.0 (5)
C57A—C5A—C6A—N1A	−173.41 (15)	C2B—N1B—C6B—C5B	2.6 (2)
C4A—C5A—C6A—C61A	−177.44 (16)	C2B—N1B—C6B—C61B	−175.34 (14)
C57A—C5A—C6A—C61A	5.4 (3)	C4B—C5B—C6B—N1B	4.3 (2)
C6B—N1B—C2B—N3B	−6.7 (2)	C57B—C5B—C6B—N1B	−169.86 (15)
C6B—N1B—C2B—C21B	172.44 (14)	C4B—C5B—C6B—C61B	−177.91 (15)
N1B—C2B—C21B—C22B	−6.1 (2)	C57B—C5B—C6B—C61B	7.9 (3)

### Hydrogen-bond geometry (Å, º)

**Table:**

*D*—H···*A*	*D*—H	H···*A*	*D*···*A*	*D*—H···*A*
N5*A*—H5*A*···N1*B*^i^	0.860 (19)	2.16 (2)	3.012 (2)	172.6 (17)
N5*B*—H5*B*···N3*A*^i^	0.91 (2)	2.54 (2)	3.403 (2)	159.7 (17)
C43*A*—H43*A*···O4*A*^ii^	0.95	2.45	3.355 (2)	159
N4*A*—H4*A*···N5*A*	0.86 (2)	2.48 (2)	3.099 (2)	130.2 (16)
N4*B*—H4*B*···N5*B*	0.87 (2)	2.31 (2)	3.021 (2)	139.0 (17)

Symmetry codes: (i) −*x*+1, −*y*+1, −*z*+1; (ii) −*x*+2, −*y*+1, −*z*+2.
